# What does scientific publishing in public health mean?

**DOI:** 10.3389/fpubh.2022.1048580

**Published:** 2022-11-29

**Authors:** Paolo Vineis

**Affiliations:** Epidemiology and Biostatistics, Imperial College London, London, United Kingdom

**Keywords:** public health, publishing, scientific peer review, paper mills, ethics

Publishing a scientific journal like Frontiers in Public Health has become more and more challenging. There are at least three reasons for the difficulties we encounter as Editors. First, scientific publishing is related to careers in many countries of the world, i.e., there is a strong pressure for professionals in biomedical sciences and public health to publish and thus increase their visibility and improve their curriculum. This problem has been boosted recently by the expansion of research funding, volume of publications and the adoption of meritocratic methods for career advancement in emerging countries such as China. The goal of publishing rapidly is starting to conflict with the main purposes of research, i.e., provide meaningful and good quality evidence that advances science and supports public health action. At this point there is an objective conflict between the two goals, the traditional one underlying the birth itself of scientific journals (make good science broadly available), and the newer one related to careers. The problem is particularly hot for open access journals like ours, that tend to be targeted by prospective authors in search for impact factor. It is urgent to address the problem of the conflict I have described, by reaffirming the scientific nature of publishing, which implies that the underlying values are quality and relevance rather than quantity and rapidity.

The second reason is the changing world of (scientific) communication, which has become itself extremely rapid and inflated. It is increasingly difficult, even above a certain qualitative threshold, to cope with the avalanche of the published papers and of those submitted for publication. The role of the Editor of a journal requires an enormous effort of comparison between the papers submitted, those already published on the same topic, the assessment of the methodology used and the relevance for the field. I do not believe that a journal is simply a repository of papers, where the readers will judge themselves about quality and relevance. I believe in the Editor's responsibility, which implies an active editorial role. A public health journal cannot simply store papers that arrive spontaneously (even after a qualitative selection), but it is supposed to guide publishing according to validity, rigor and relevance. Otherwise, the journal may become overwhelmed by misleading articles that miss the opportunity of advancing the science and supporting action; something that may be already happening. The signal should emerge clearly from noise, which is not always the case in a world in which controversial and polarizing content is fueled by commercial incentives.

In this context there is a third, practical problem that explains why publishing a journal is hard, the role of reviewers. It has become notoriously difficult to find good reviewers, or reviewers at all. Our journal struggles with the multiple refusals by colleagues invited to peer review manuscripts submitted in their field. This failure in finding (good) reviewers means that less experienced ones are involved, hampering the quality of reviews. In principle reviewing a paper can be done by a junior colleague, and this has an educational component; but this should occur under a senior person's supervision and responsibility, which happens rarely. Incentives to peer review papers are limited, essentially recognition in early stages of career. It is more rewarding publishing than reviewing, which creates an asymmetry.

The reality is that a journal like ours can see a large number of manuscripts sometimes with aggressive accompanying messages that imply rapid and acritical acceptance, almost a claim to the right of publishing (i.e., a misinterpretation of the role of journal). We encounter difficulties in finding adequate reviewers, and we thereby risk falling into Type I and Type II errors, i.e., accepting wrong and poor papers or, vice versa, rejecting by mistake important contributions. By the way, it may even be in the (misinterpreted) interest of the journal falling into Type I errors, because controversial papers can be frequently cited and increase visibility and the Impact Factor. For example, as my colleague Marc Struelens and I have already stressed in a previous Perspective, research on COVID-19 has been so massive that it has been difficult to avoid both types of error (1). For example, we stressed the limitations of using geographic data to draw inferences on the effectiveness of containment measures for COVID-19, but nevertheless Frontiers in Public Health has published such a paper after our Perspective (2). The huge flow of papers is accompanied by very unfortunate side effects, which affect only a small minority of submissions but are however extremely serious, like plagiarism or evidence fabrication (in the more general wave of paper mills).

Of course, all these problems are well known to journal Editors, and codes of practice have been developed, like COPE (https://publicationethics.org/). However, my feeling is that the acceleration of research and its increasing amount has made the situation worse than in the past.

Is it all negative? Of course not. As for many phenomena of the world we are currently living in, there are new and exciting perspectives together with the limitations and drawbacks I have described. What our publishing work reflects is a great expansion of the research community, which is quantitative but also qualitative: a large number of new colleagues are inter-connected, particularly from emerging countries and low-income countries. This corresponds to a less Euro-centric and Americo-centric view of science and public health, and with publications that in tendency reflect the problems of the world more faithfully than in journals of the past dominated by a limited number of institutions. However, to take advantage of these positive features of the expansion of borders, research communities, languages and skills, we need to reaffirm the key values of scientific publishing and find the most adequate procedures to transfer them into practice.

## Author contributions

The author confirms being the sole contributor of this work and has approved it for publication.

## Conflict of interest

The author declares that the research was conducted in the absence of any commercial or financial relationships that could be construed as a potential conflict of interest.

## Publisher's note

All claims expressed in this article are solely those of the authors and do not necessarily represent those of their affiliated organizations, or those of the publisher, the editors and the reviewers. Any product that may be evaluated in this article, or claim that may be made by its manufacturer, is not guaranteed or endorsed by the publisher.
